# Supplementary material for: Plasma cell-free RNA signatures of inflammatory syndromes in children
Source: Proc Natl Acad Sci U S A. 2024 Sep 6;121(37):e2403897121. doi: 10.1073/pnas.2403897121 (PMC11406294; doi:10.1073/pnas.2403897121)
Supplement: Supplementary file 1 — Appendix 01 (PDF) [file pnas.2403897121.sapp.pdf]

PNAS

Supporting Information for
Plasma Cell-Free RNA Signatures of Inflammatory Syndromes in Children

Authors: Conor J. Loy[1], Venice Servellita[2], Alicia Sotomayor-Gonzalez[2], Andrew Bliss[1], Joan Lenz[1], Emma Belcher[1], Will Suslovic[3], Jenny Nguyen[2], Meagan E. Williams[3], Miriam Oseguera[2], Michael A. Gardiner[4], Pediatric Emergency Medicine Kawasaki Disease Research Group (PEMKDRG), The CHARMS Study Group, Jong-Ha Choi[5], Hui-Mien Hsiao[5], Hao Wang[6], Jihoon Kim[7], Chisato Shimizu[6], Adriana Tremoulet[4,6], Meghan Delaney[3], Roberta L. DeBiasi[3], Christina A. Rostad[5], Jane C. Burns[4,6]*, Charles Y. Chiu[2,8,9]*, Iwijn De Vlaminck[1]*

Jane Burns
Email: jcburns@health.ucsd.edu

Charles Chiu
Email: charles.chiu@ucsf.edu

Iwijn De Vlaminck
Email: vlaminck@cornell.edu

This PDF file includes:

Figures S1 to S3
Tables S1 to S3
Legends for Files S1 to S7
SI References
Contributor Information

Other supporting materials for this manuscript include the following:

Datasets S1 to S7

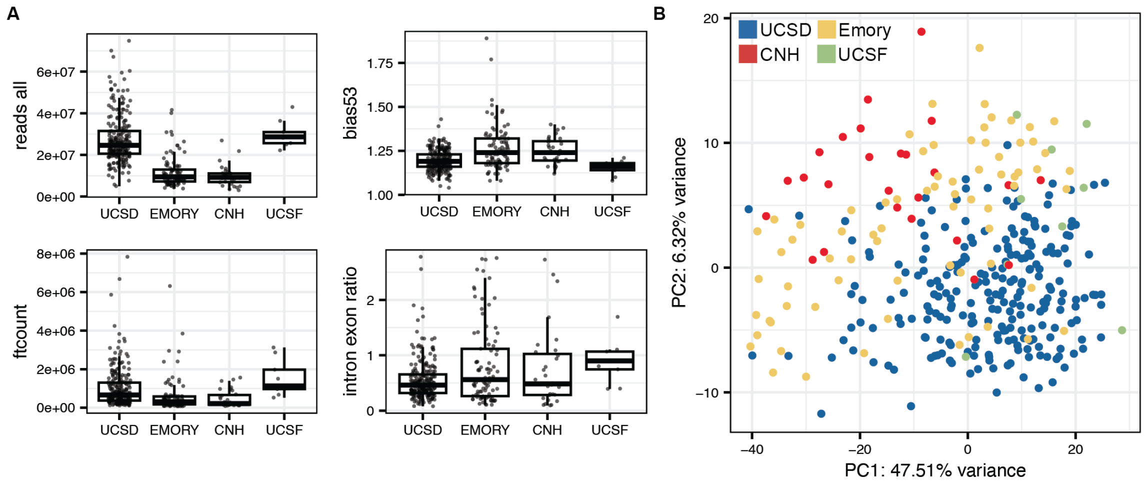

Fig. S1. cfRNA Quality Control

(A) Sample sequencing metrics, including all sequenced reads, 5'-3' bias, number of feature counts, and intron to exon ratio for all samples split by hospital of origin.

(B) Principal component analysis on variance stabilization transformed counts of all samples using the top 500 most variable genes.

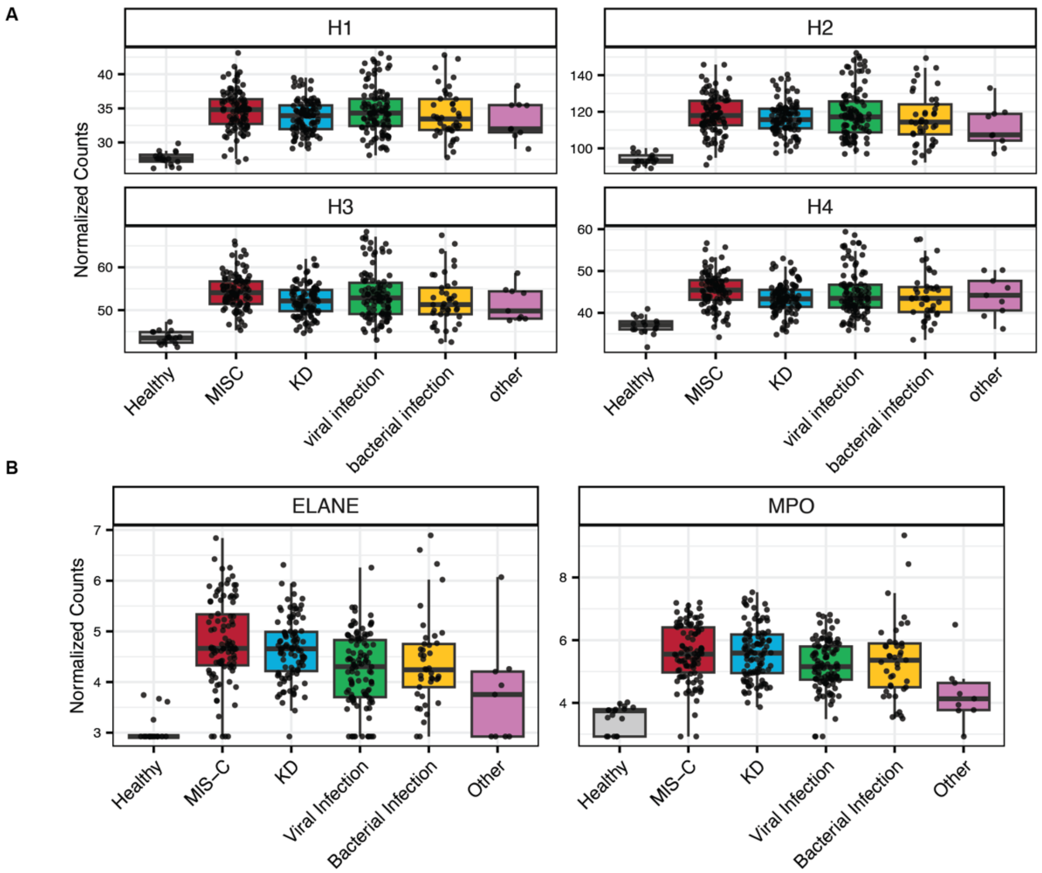

Fig. S2. cfRNA molecules elevated in disease

(A) Sum variance stabilization transformation counts of histone protein transcripts found to be differentially abundant in healthy and all other conditions. Counts grouped by the histone protein the differentially abundant cfRNA code for.

(B) Variance stabilization transformation counts of ELANE and MPO transcripts.

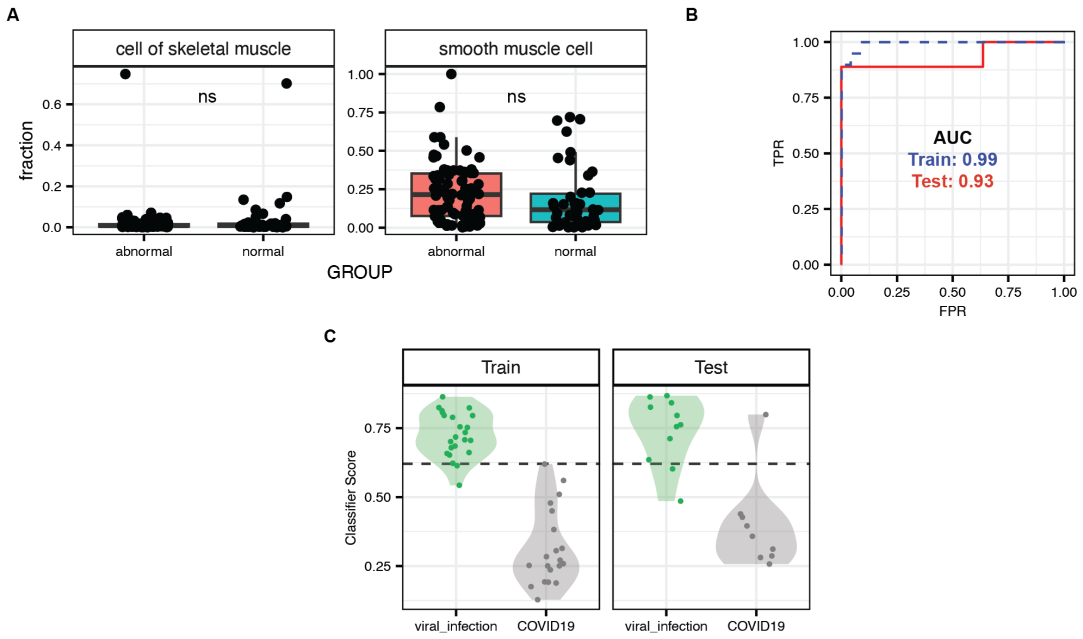

Fig. S3. Characterization of disease

(A) Cell type of origin fractions of skeletal and smooth muscle cells in patients with abnormal and normal cardiac function. Significance calculated with a Wilcoxon test and p-values adjusted using the BH method.

(B) ROC-AUC plot and

(C) classification scores of the train and test sets for classifying COVID-19 vs other viral infections as modeled by a GLMNET LASSO.

Variables	Group	MIS-C	KD	Viral Infection	Bacterial Infection	Other	Healthy
**n**		97	101	105	41	9	17
**Origin, n (%)**	UCSD	42 (43.30)	99 (98.02)	71 (67.62)	25 (60.98)	0 (0.00)	0 (0.00)
	Emory	43 (44.33)	1 (0.99)	29 (27.62)	0 (0.00)	3 (33.33)	17 (100.00)
	CNH	12 (12.37)	1 (0.99)	5 (4.76)	7 (17.07)	6 (66.67)	0 (0.00)
	UCSF	0 (0.00)	0 (0.00)	0 (0.00)	9 (21.95)	0 (0.00)	0 (0.00)
**Race, n (%)**	Asian	1 (1.03)	17 (16.83)	6 (5.71)	1 (2.44)	0 (0.00)	0 (0.00)
	Hispanic or Latino	23 (23.71)	31 (30.69)	28 (26.67)	5 (0.00)	0 (0.00)	0 (0.00)
	Black / African American	39 (40.21)	5 (4.95)	22 (20.95)	4 (9.76)	3 (33.33)	3 (17.65)
	Caucasian	14 (14.43)	18 (17.82)	23 (21.90)	13 (31.71)	4 (44.44)	7 (41.18)
	American Indian / Alaska Native	0 (0.00)	1 (0.99)	0 (0.00)	0 (0.00)	0 (0.00)	0 (0.00)
	Native Hawaiian or Other Pacific Islander	0 (0.00)	1 (0.99)	0 (0.00)	0 (0.00)	0 (0.00)	0 (0.00)
	Other	1 (1.03)	2 (1.98)	1 (0.95)	2 (4.88)	0 (0.00)	0 (0.00)
	More than one race	15 (15.46)	26 (25.74)	20 (19.05)	11 (26.83)	2 (22.22)	7 (41.18)
**Sex, n (%)**	Male	71 (73.20)	57 (56.44)	57 (54.29)	25 (60.98)	6 (66.67)	6 (35.29)
	Female	26 (26.80)	44 (43.56)	48 (45.71)	16 (39.02)	3 (33.33)	11 (64.71)
**ICU, n (%)**	Yes	69 (71.13)	3 (2.97)	14 (13.33)	7 (17.07)	3 (33.33)	0 (0.00)
	No	28 (28.87)	98 (97.03)	91 (86.67)	34 (82.93)	6 (66.67)	17 (100.00)

Table S1. Sample Cohort Overview

Gene ID	Gene Name	Gene Type
ENSG00000265972.6	TXNIP	protein coding
ENSG00000116539.14	ASH1L	protein coding
ENSG00000119801.13	YPEL5	protein coding
ENSG00000169564.7	PCBP1	protein coding
ENSG00000081320.11	STK17B	protein coding
ENSG00000115993.13	TRAK2	protein coding
ENSG00000187514.17	PTMA	protein coding
ENSG00000201822.1	RNA5SP149	rRNA pseudogene
ENSG00000172493.23	AFF1	protein coding
ENSG00000137460.10	FHDC1	protein coding
ENSG00000197043.14	ANXA6	protein coding
ENSG00000096060.15	FKBP5	protein coding
ENSG00000096384.20	HSP90AB1	protein coding
ENSG00000131018.25	SYNE1	protein coding
ENSG00000184863.11	RBM33	protein coding
ENSG00000026025.16	VIM	protein coding
ENSG00000150593.18	PDCD4	protein coding
ENSG00000026508.20	CD44	protein coding
ENSG00000174718.12	RESF1	protein coding
ENSG00000183283.16	DAZAP2	protein coding
ENSG00000103187.8	COTL1	protein coding
ENSG00000108518.8	PFN1	protein coding
ENSG00000167658.16	EEF2	protein coding
ENSG00000123144.11	TRIR	protein coding
ENSG00000087086.15	FTL	protein coding

Supplementary Table 2. Gene panel for KD vs MIS-C prediction

Comparison	Cohort	AUC	Sensitivity	Specificity
KD vs Bacterial Infection	train	0.99	0.94	1
	test	0.87	0.97	0.71
KD vs Viral Infection	train	0.99	0.97	0.96
	test	0.93	0.94	0.79
MIS-C vs Bacterial Infection	train	1	0.98	1
	test	0.94	0.91	0.79
MIS-C vs Viral Infection	train	1	1	0.99
	test	0.97	0.84	0.94
MIS-C vs KD	train	1	0.98	1
	test	0.99	0.91	0.94
Viral Infection vs Bacterial Infection	train	0.97	0.96	0.85
	test	0.86	0.88	0.71
KD vs All	train	-	1	1
	test	-	0.84	0.9
MIS-C vs All	train	-	1	1
	test	-	0.91	0.96
Bacterial Infection vs All	train	-	1	1
Bacterial Infection vs All	test	-	0.43	0.95
Viral Infection vs All	train	-	1	1
Viral Infection vs All	test	-	0.82	0.92

Supplementary Table 3. Model performance metrics

Dataset S1 (separate file). Bacterial and viral infection sample descriptions.

Dataset S2 (separate file). DESeq2 output table from each healthy vs disease comparison.

Dataset S3 (separate file). Qiagen IPA output of the DAGs for all conditions vs healthy (BH adjusted p-value <0.05). Average adjusted p-value and average log2FoldChange input.

Dataset S4 (separate file). DESeq2 output table from KD vs MIS-C comparison using the training data set.

Dataset S5 (separate file). DESeq2 output tables for each 1 vs 1 comparison using the training data set.

Dataset S6 (separate file). List of 109 genes chosen and used by the multiclass algorithm.

Dataset S7 (separate file). Consolidated model and cell-type of origin z-scores for all samples from the test set. Samples bordered in red were incorrectly classified, samples bordered in black were correctly classified.

Contributor Information

Pediatric Emergency Medicine Kawasaki Disease Research Group (PEMKDRG) Members:
Lukas Austin-Page, MD
Amy Bryl, MD
Joelle Donofrio-Ödmann, MD
Atim Ekpenyong, MD
David Gutglass, MD
Scott Herskovitz, MD
Paul Ishimine, MD
John Kanegaye, MD
Margaret Nguyen, MD
Mylinh Nguyen, MD
Kristy Schwartz, MD
Stacey Ulrich, MD
Tatyana Vayngortin, MD
Elise Zimmerman, MD
* All PEMKDRG Members are at Rady Children's Hospital, San Diego, CA, USA

Characterization of Multisystem Inflammatory Syndrome in Children (CHARMS) Members:
Jocelyn Ang, MD – Children's Hospital of Michigan
Margalit Rosenkranz, MD – UPMC Children's Hospital of Pittsburgh
Joseph Bochini, MD – Knighton Children's Health Tulane University
Michelle Sykes, MD – Valley Children's Healthcare
Lerraughn Morgan, MD – Valley Children's Healthcare
Laura D'Addese, MD – Joe Dimaggio Children's Hospital (FL)
Maria Pilar Gutierrez, MD – Joe Dimaggio Children's Hospital (FL)
