# Supplementary figures and images for: Plasma cell-free RNA signatures of inflammatory syndromes in children
Source: Proc Natl Acad Sci U S A. 2024 Sep 6;121(37):e2403897121. doi: 10.1073/pnas.2403897121 (PMC11406294; doi:10.1073/pnas.2403897121)

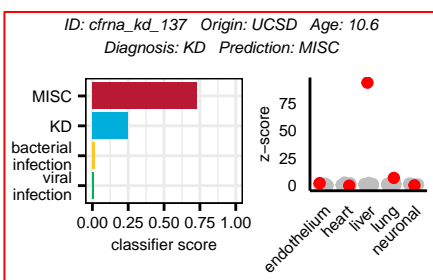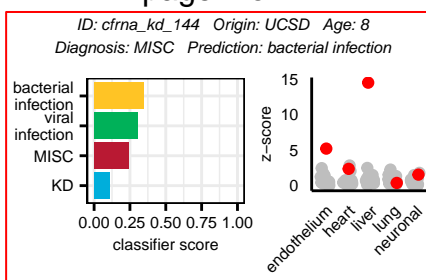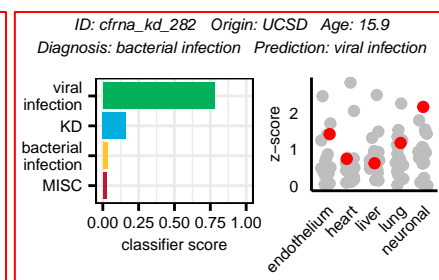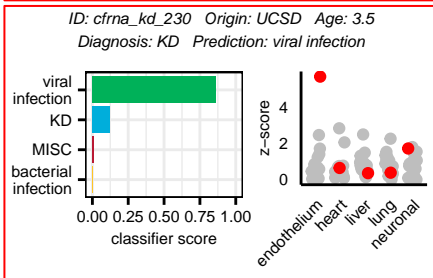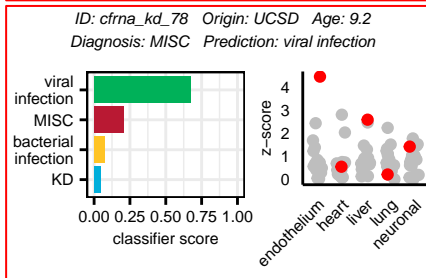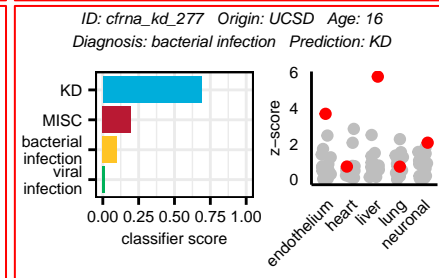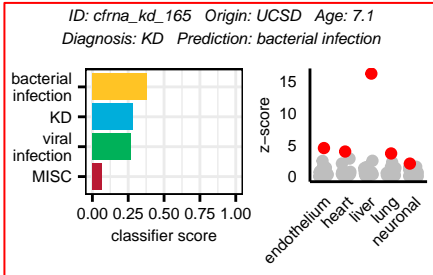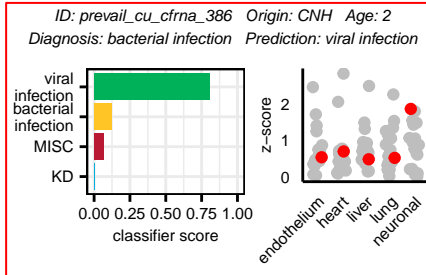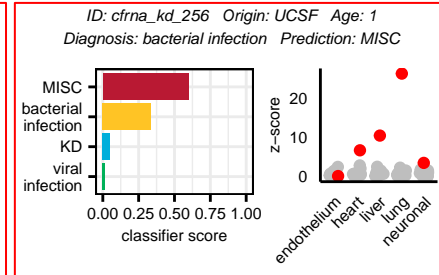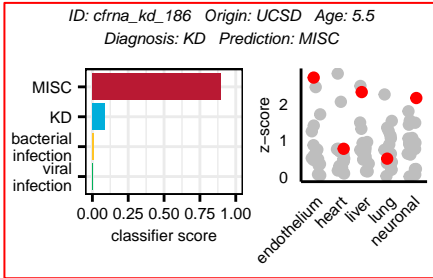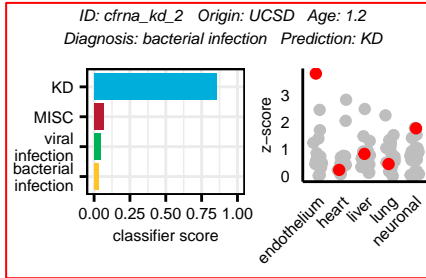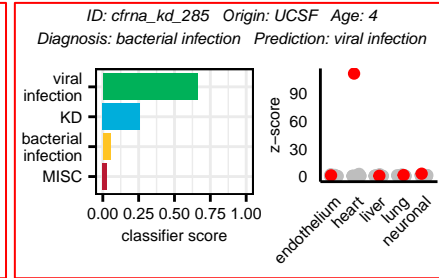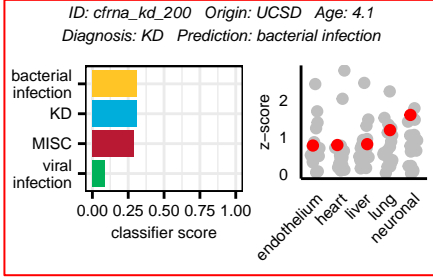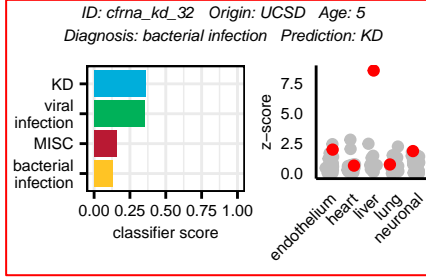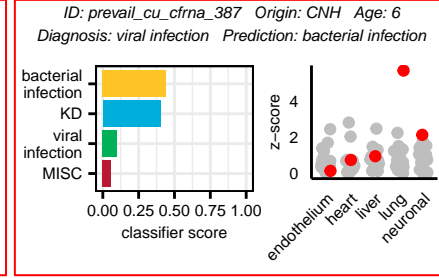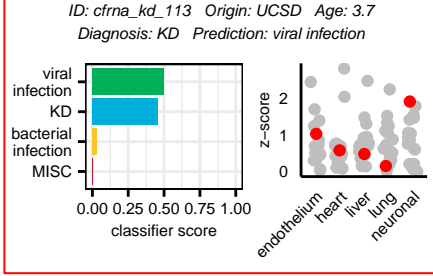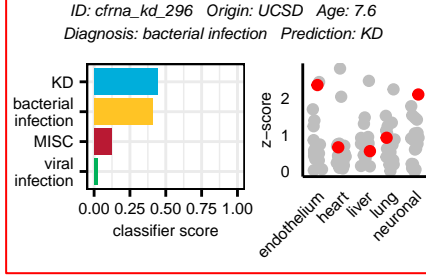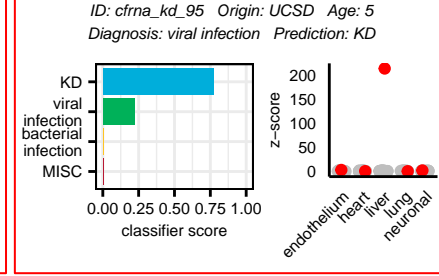

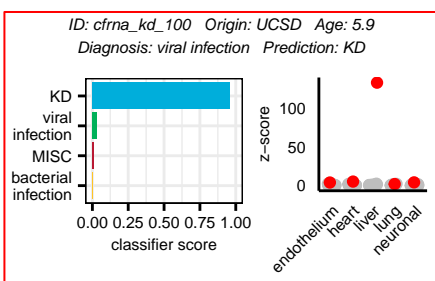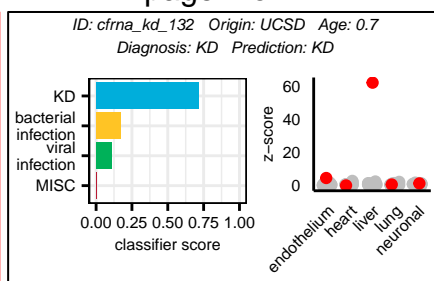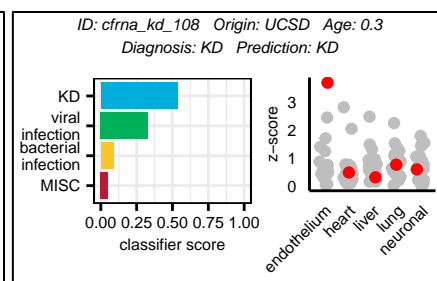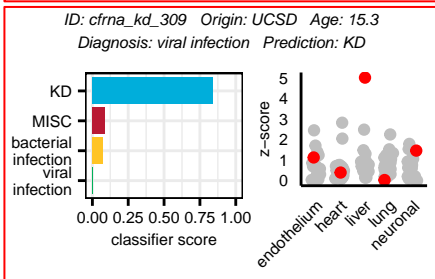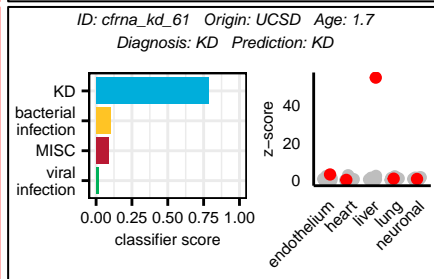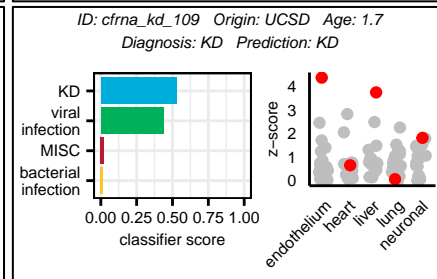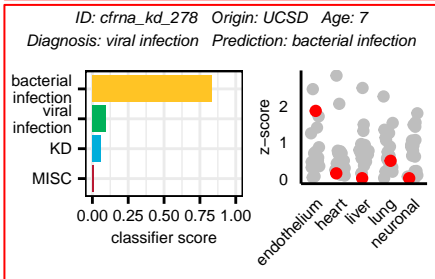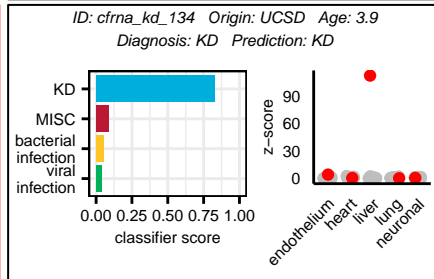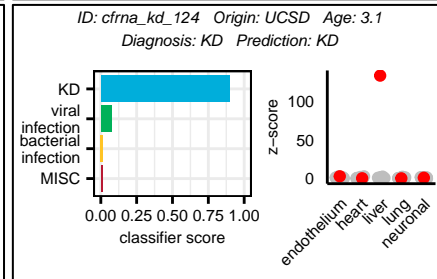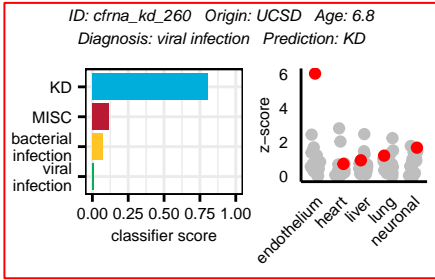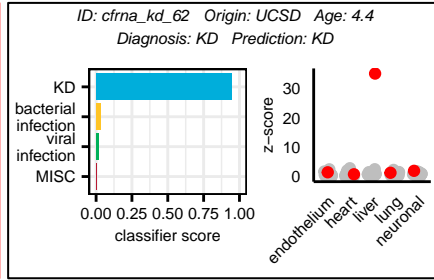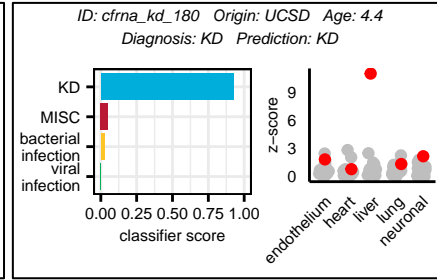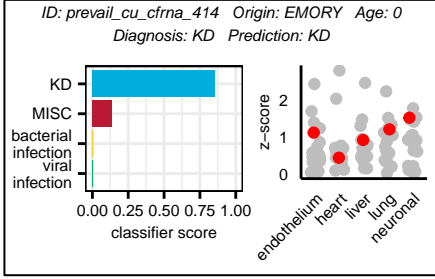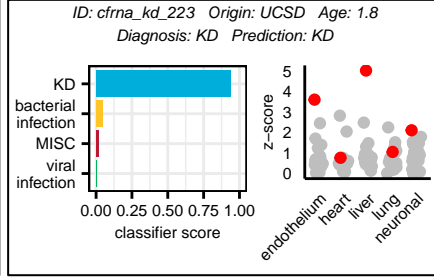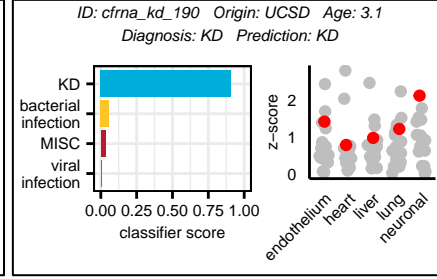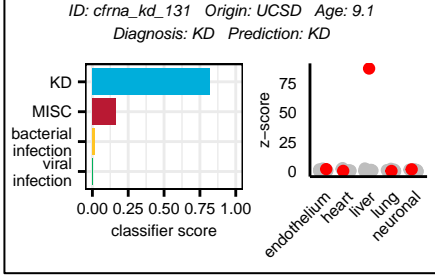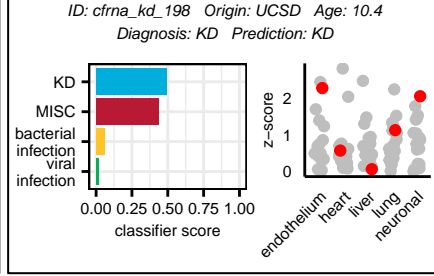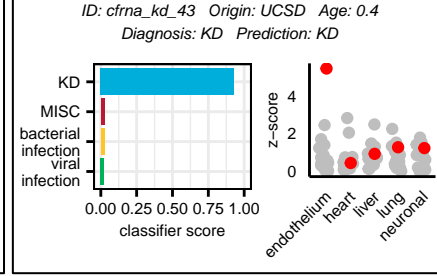

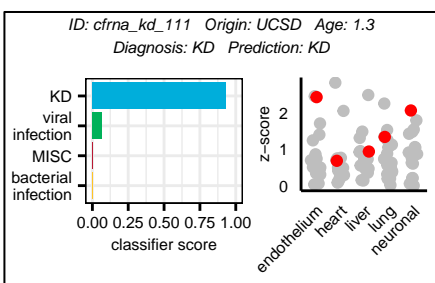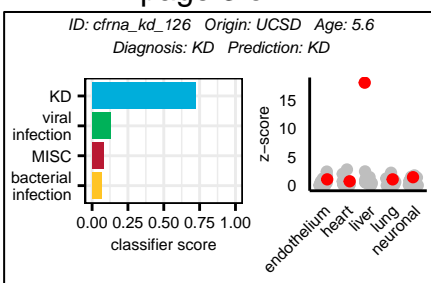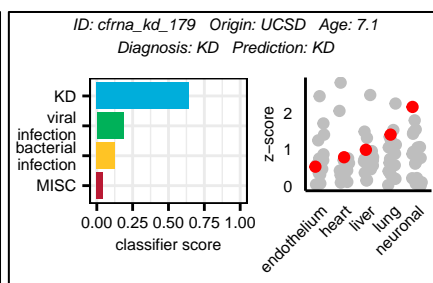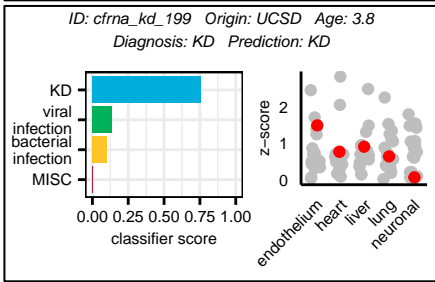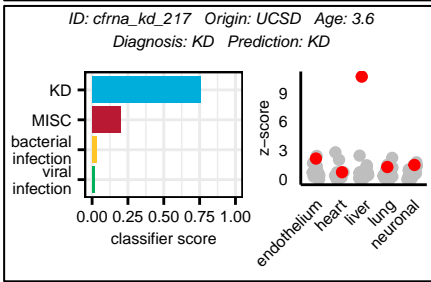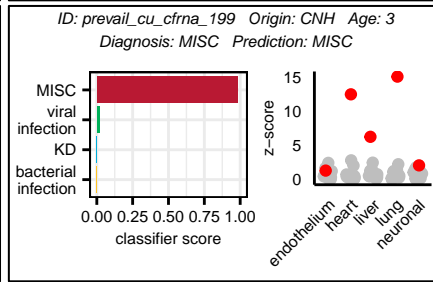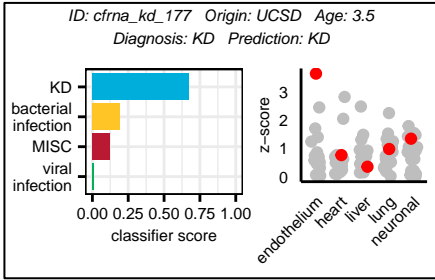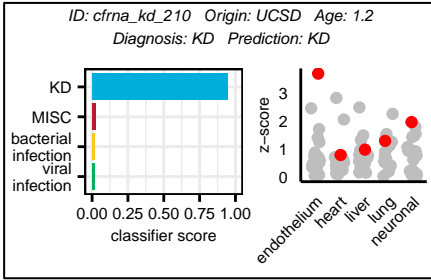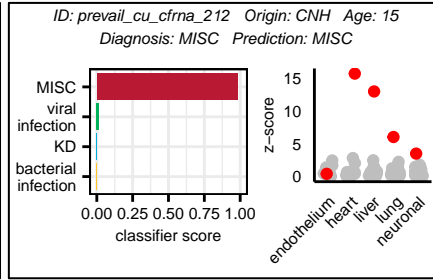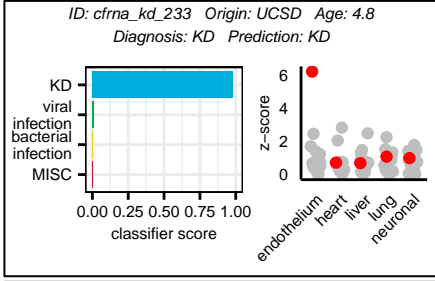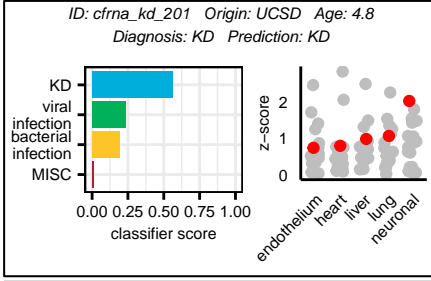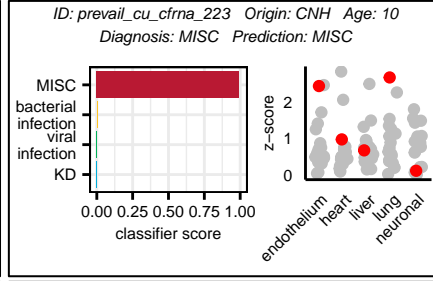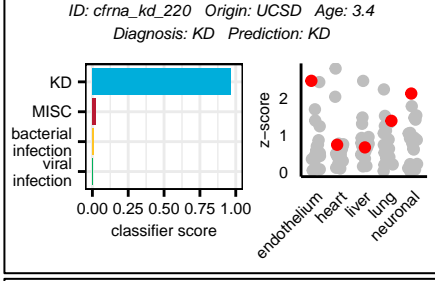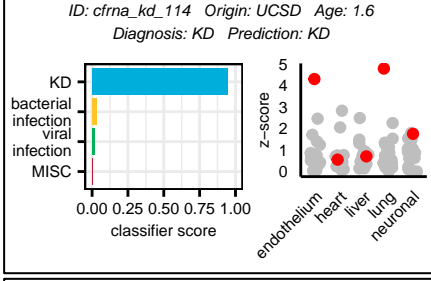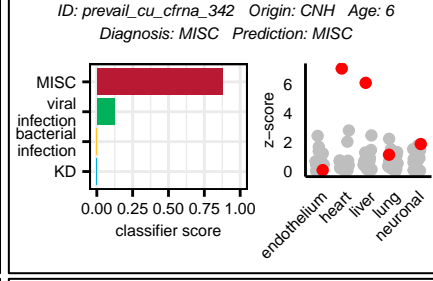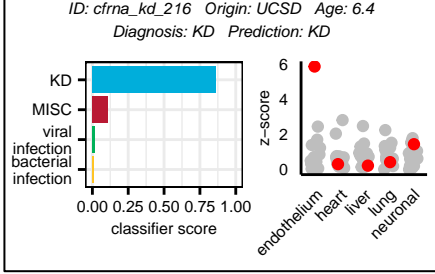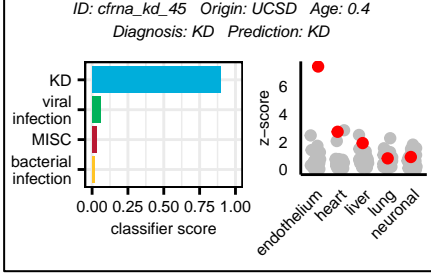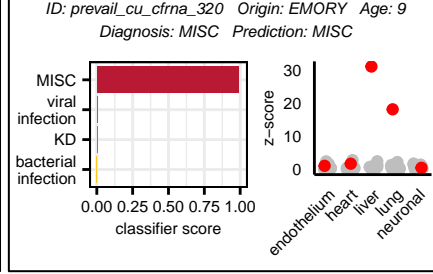

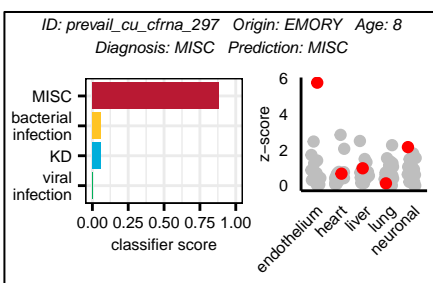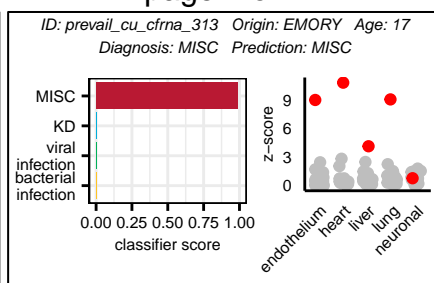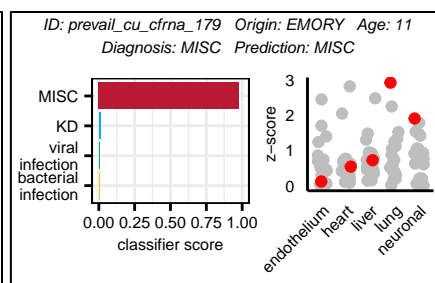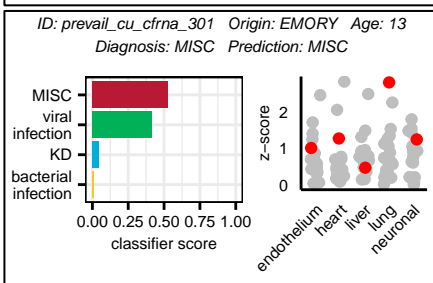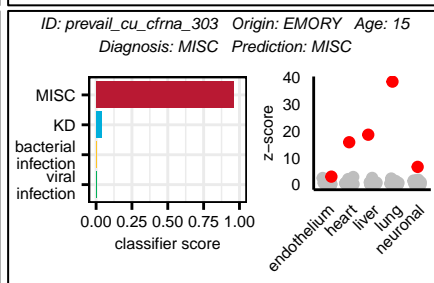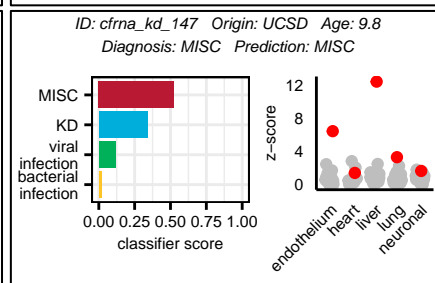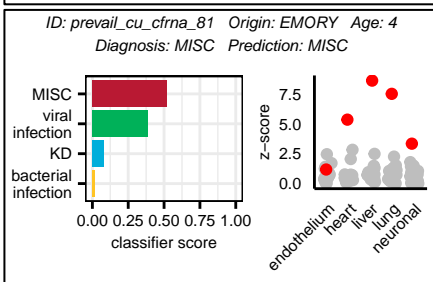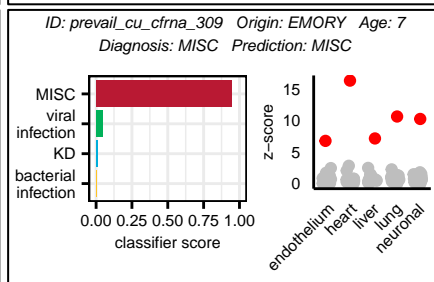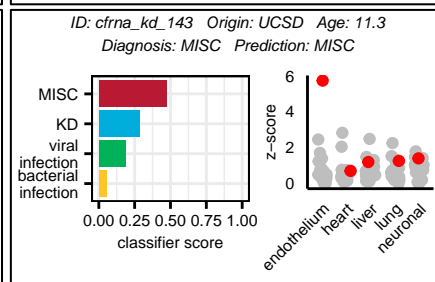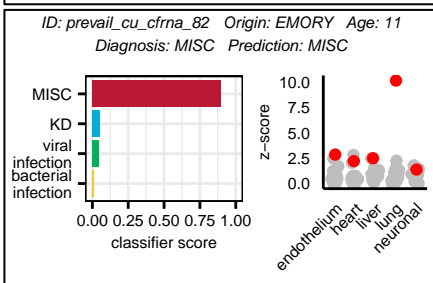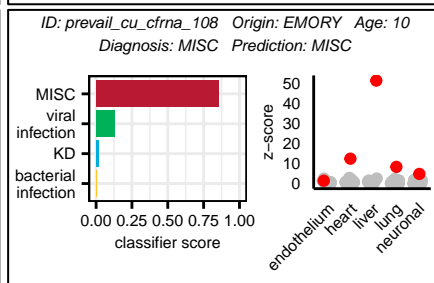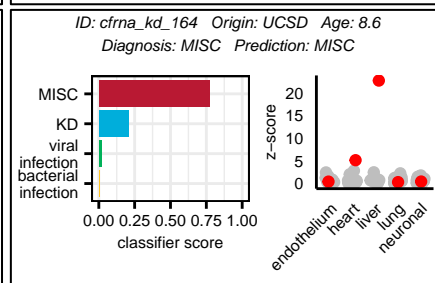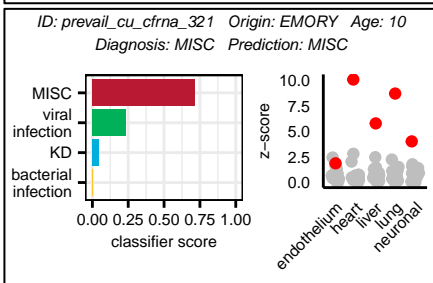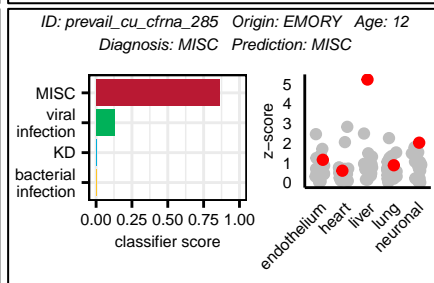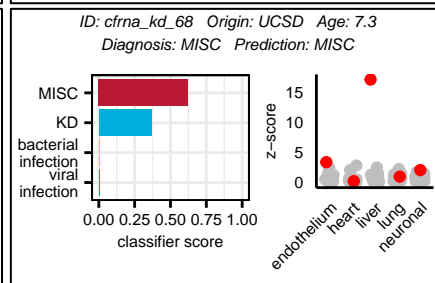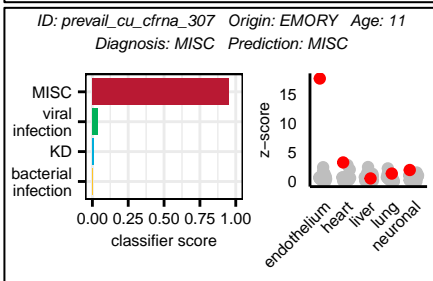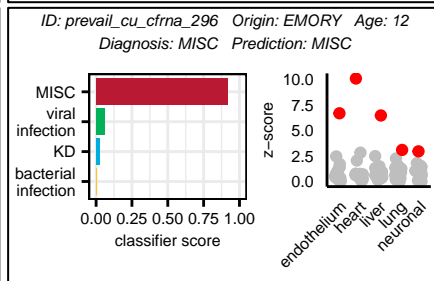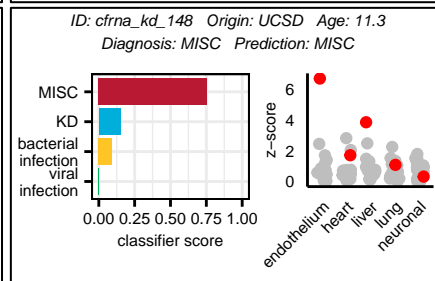

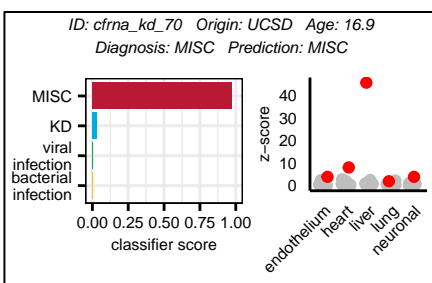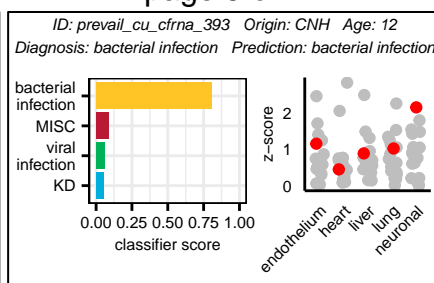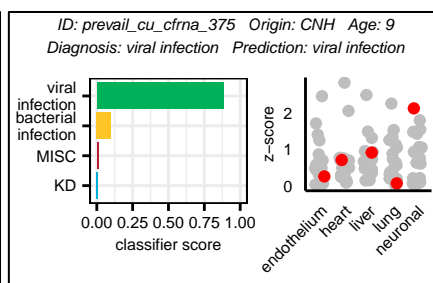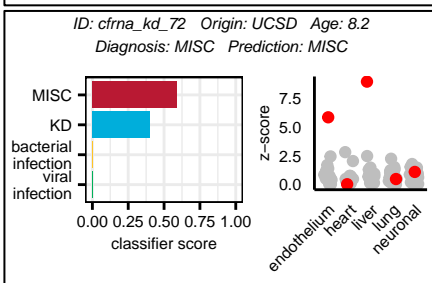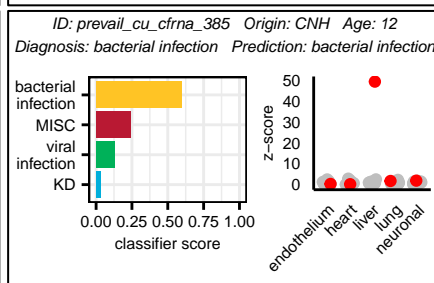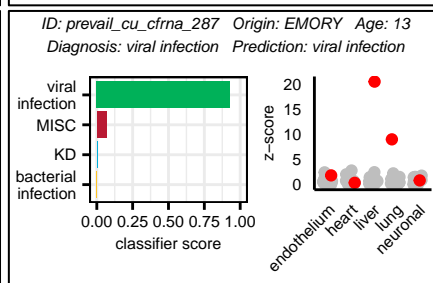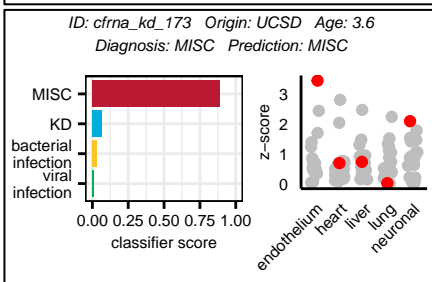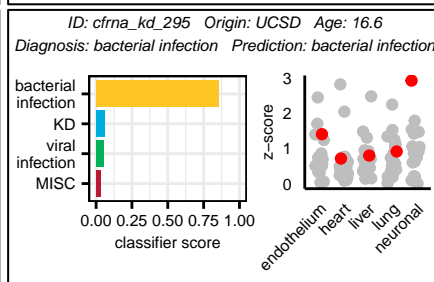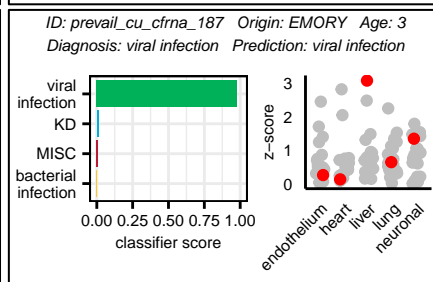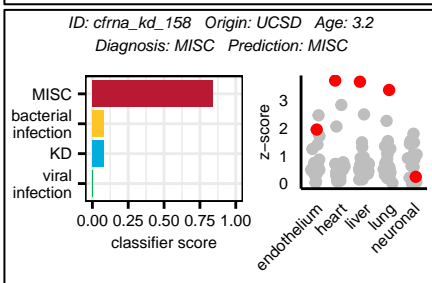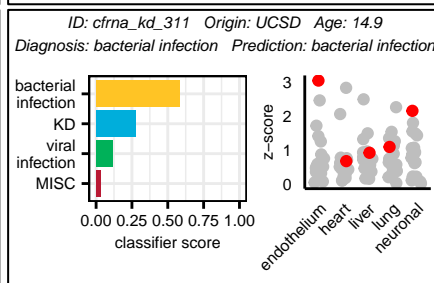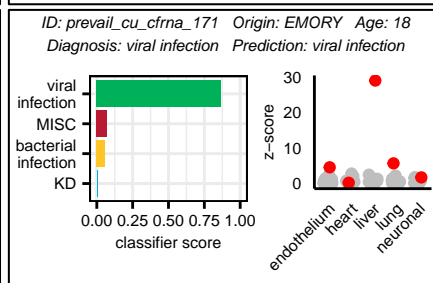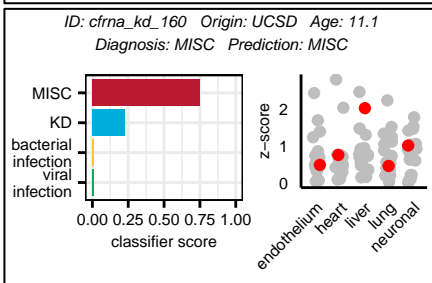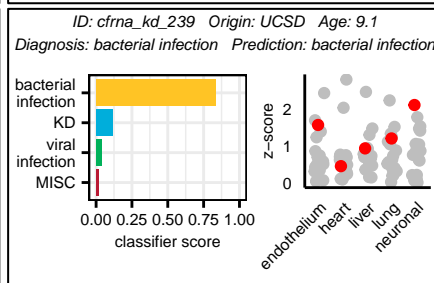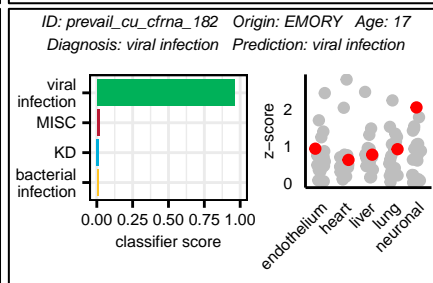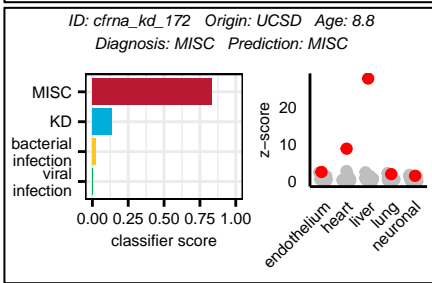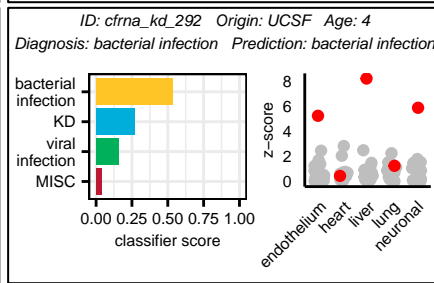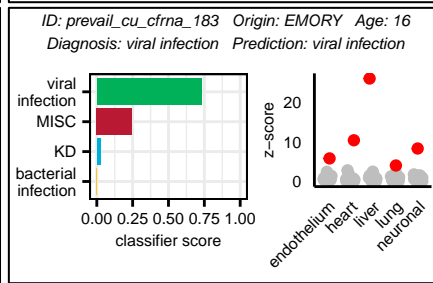

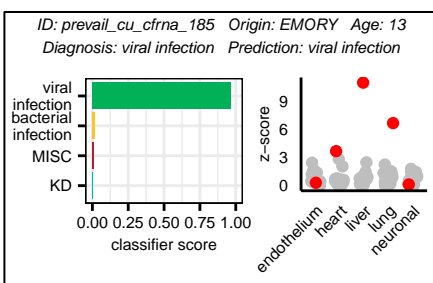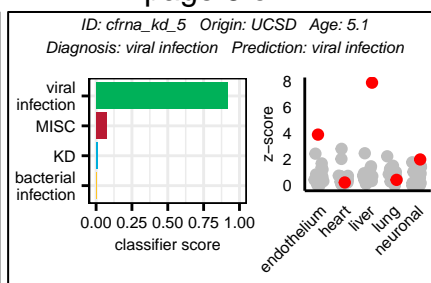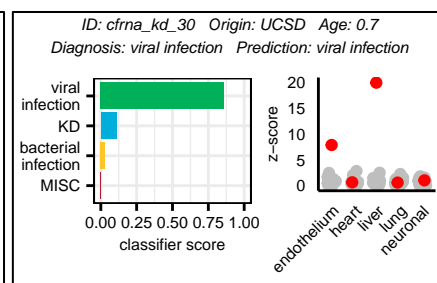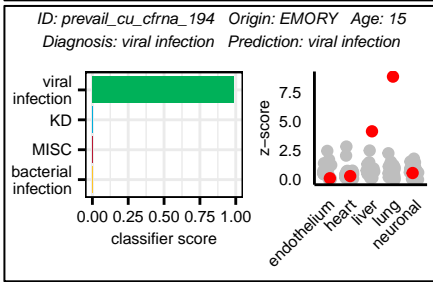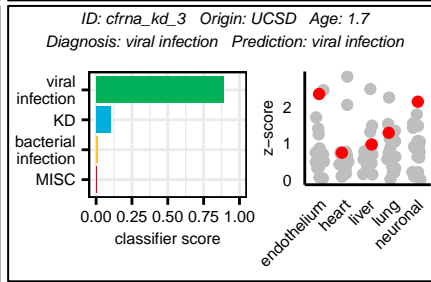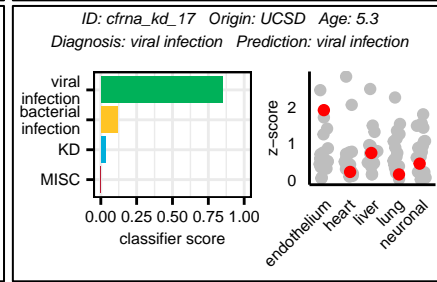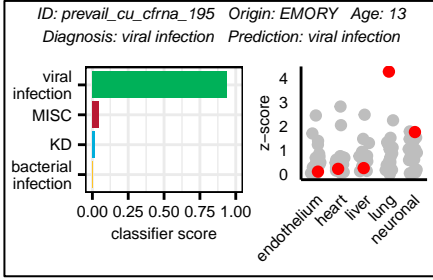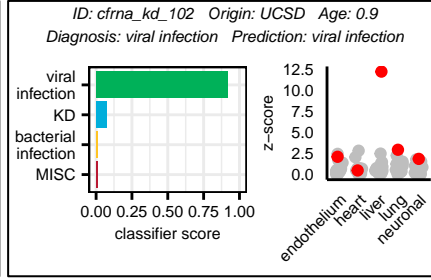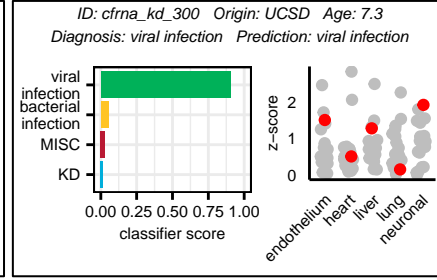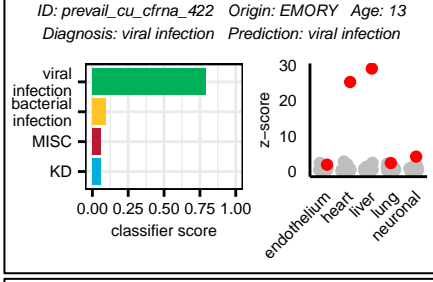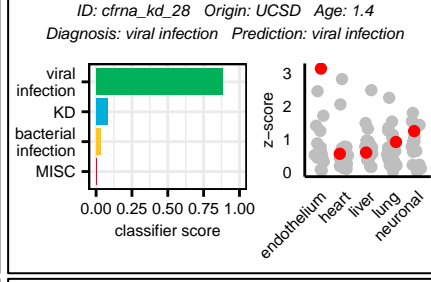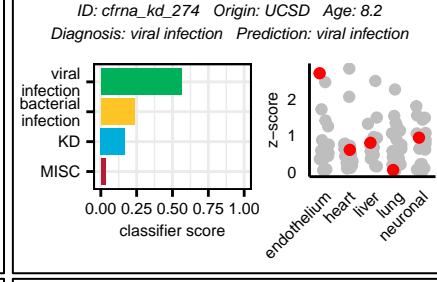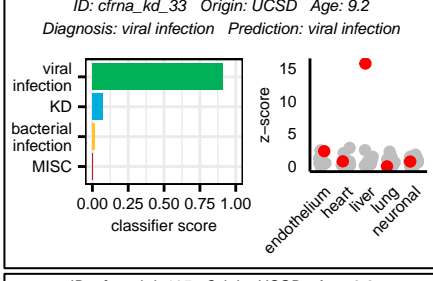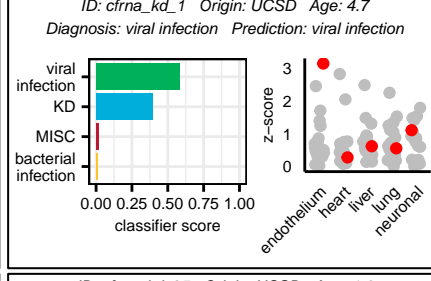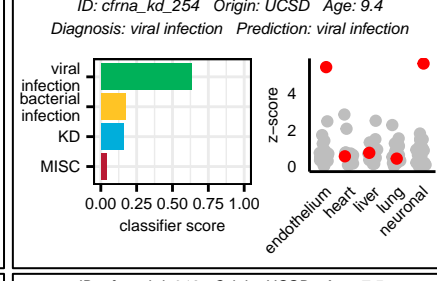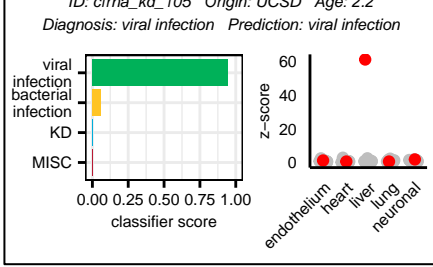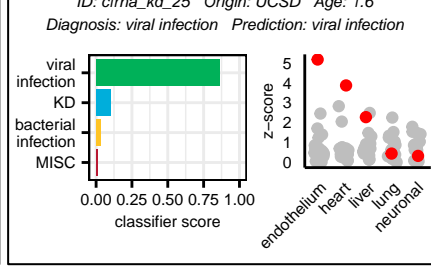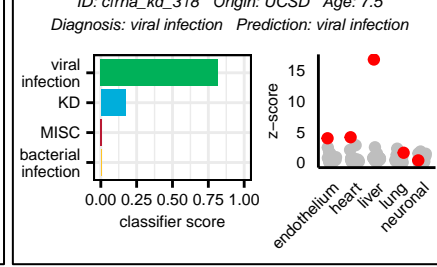

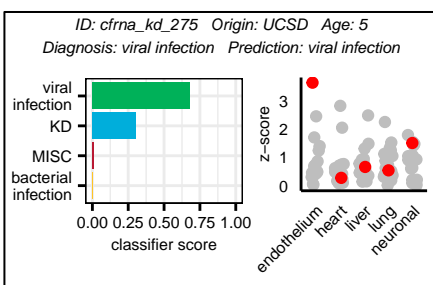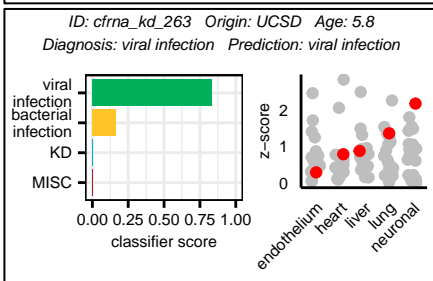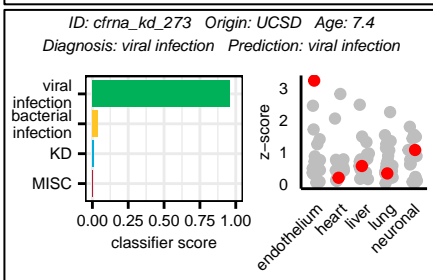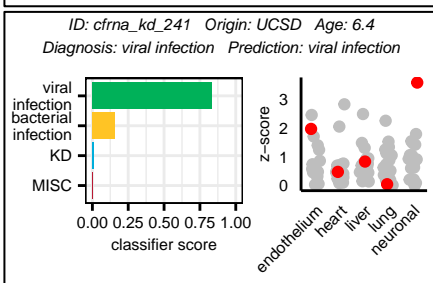

Supplement: Supplementary file 8 — Dataset S07 (PDF) [file pnas.2403897121.sd07.pdf]
